# Evidence of cellular adaptations in a fungal cultivar promoting resource exchange with leafcutter ant farmers

**DOI:** 10.1098/rsbl.2025.0259

**Published:** 2025-10-01

**Authors:** Ayoub Stelate, Jonathan Zvi Shik

**Affiliations:** ^1^ Section for Ecology and Evolution, Department of Biology, University of Copenhagen, Copenhagen 2100, Denmark; ^2^ Smithsonian Tropical Research Institute, Balboa, Ancon 0843-03092, Panama

**Keywords:** *Atta*, cytosol, fungal hyphae, gongylidia, *Leucoagaricus gongylophorus*, mutualism, pH, reactive oxidative species, vacuole

## Abstract

Leafcutter farming systems are ant–fungus mutualisms whose ecological success hinges on differentiation of fungal hyphae into swollen cells called gongylidia that ants consume. While gongylidium cells are unique signatures of coevolved crop domestication, their cell biology is poorly understood. Each gongylidium cell contains a large vacuole that is thought to protectively store plant degradation enzymes that ants ingest and vector unharmed in faecal droplets back to the fungus. We hypothesized that enzyme storage requires gongylidium vacuoles to have distinct levels of pH and reactive oxygen species (ROS) compared to the vacuoles of undifferentiated hyphae that likely degrade cellular waste. We used live-cell fluorescence microscopy of fungal isolates with targeted probes to first show that both cell types had vacuoles with lower pH than the surrounding cytosol. In contrast, while hyphal vacuoles stored ROS, gongylidium vacuoles excluded these potentially harmful molecules. These findings suggest derived cellular adaptations in a mutualistic fungus where gongylidia protect ant-vectored enzymes through specialized subcellular ROS compartmentalization.

## 1. Introduction

Mutualisms between fungi and animals are diverse and ecologically important [1]. Although animal partners often depend on fungus-derived metabolites, the cellular basis of this biochemical exchange remains poorly understood. We propose that the altruistic transfer of resources to animal partners has favoured the evolution of specialized cellular adaptations in fungal mutualists [2,3]. In turn, these cellular adaptations can provide evolutionary insights into the resource exchange mechanisms promoting mutualism stability, especially when fluctuating environments change trade incentives.

We explored these mechanisms in leafcutter ant farming systems (*Atta*, *Acromyrmex*) where up to millions of ants cultivate the fungus *Leucoagaricus gongylophorus* (order: Agaricales, family: Agaricaceae) in monoculture [4,5]. Ants forage plant material [6] and provision it to the fungus, which converts it into an optimized diet sustaining the colony [7–9]. While ant adaptations maximizing cultivar performance have been well characterized [10–13], the reciprocal crop productivity adaptations in the fungal cultivar are less resolved. The fungus is known^3^ to produce enzymes that degrade and detoxify ant-foraged plant fragments [10,14–19] and to synthesize essential nutrients like arginine for ant farmers [20].

The cultivar also exhibits signatures of coevolved crop domestication. First, their genomes are rich in transposable elements and have expanded biosynthetic gene clusters [3]. These traits reflect relaxed purifying selection and metabolic specialization under long-term cultivation. Second, the fungal cells are multinucleate and exhibit a form of fungal polyploidy that may enhance biosynthetic output [21]. Polyploidy is a common feature of plant crops domesticated by humans [22]. Third, the fungus produces specialized edible cells called gongylidia that appear to be unique in the fungal kingdom and are analogous to the engorged fruits produced by other domesticated fruit crops [2].

Gongylidia are swollen cells that differentiate from surrounding hyphae and concentrate enzymes, nutrients and possibly signalling compounds [23–25]. Each gongylidium cell houses a large vacuole, which is an organelle known in fungi to facilitate transport, storage and cellular homeostasis [26,27]. Ants assimilate gongylidium nutrients while simultaneously passing enzymes unaltered through their digestive systems. The ants subsequently vector these enzymes back to the fungus garden through faecal fluids to promote degradation of newly deposited plant material [9,18,28]. While the functionality of ant-vectored enzymes has been confirmed [19,29], it remains unknown whether gongylidium cells exhibit physiological modifications relative to undifferentiated hyphae that enable protective enzyme storage before ant ingestion.

We tested the hypothesis that gongylidium cells exhibit physiological differences coinciding with their morphological differentiation from typical hyphal cells. We first measured cytosolic and vacuolar pH in live fungal cells, as pH is a key intracellular factor influencing metabolism and enzyme function [30–32]. We then measured reactive oxygen species (ROS), since this by-product of metabolism and enzymatic reactions can cause oxidative stress that damages cells [33,34]. Although low ROS levels would be expected since they support signalling and various physiological processes, excessive ROS are often sequestered in vacuoles to prevent damage to cells [35–37]. Here, we predicted that the protective storage of ant-targeted metabolites would preclude ROS sequestration in gongylidium vacuoles. We tested these hypotheses using live-cell fluorescence microscopy with pH-sensitive and ROS-specific dyes.

## 2. Material and methods

### (a) Biological material

A colony of the leafcutter ant *Atta colombica* (TLP-20211014-11) was collected from Soberanía National Park in Panama and maintained under controlled laboratory conditions at the University of Copenhagen. The colony was kept at 25°C and 70% relative humidity with minimal exposure to daylight. The fungal symbiont, *Leucoagaricus gongylophorus*, was isolated from the colony and cultured axenically in five separate 90 mm Petri dishes, each containing 20 ml of potato dextrose agar [7]. Cultures were incubated in the dark at 25°C for four weeks.

### (b) Measurement of intracellular pH

Fungal samples were incubated on Petri dishes with pH-sensitive fluorescent probes under controlled conditions. Cytosolic pH was assessed using 5 µM of carboxyl SNARF-1 acetoxymethyl ester (SNARF-1AM; ThermoFisher) [38] diluted from a 2 mM DMSO stock. The working solution contained 5 µM carboxyl SNARF-1AM, 0.01% Pluronic F-127 and 0.1% DMSO in phosphate-buffered saline (PBS). Fungal cells were incubated in this solution in the dark at 25°C for 20 min and then imaged. Cytosolic pH was quantified using ratiometric analysis of carboxyl SNARF-1 fluorescence, with fluorescence emission recorded at 585 nm (orange; indicative of more acidic pH) and 640 nm (±10 nm) (red; indicative of more neutral to basic pH). The fluorescence intensity ratio was calibrated *in vivo* within a pH range of 6.0−8.5 following the manufacturer’s manual.

Vacuolar pH was measured using 5 µM pHrodo Green AM (ThermoFisher) [39] prepared from a 10 mM DMSO stock solution. Samples were incubated with this dye under the same conditions. Vacuolar pH was quantified using fluorescence intensity at 535 nm (±10 nm) within selected imaged regions that avoided background fluorescence. We used the carboxyl SNARF-1AM probe for the cytosol and the pHrodo probe for the vacuole because they exhibit different optimal localization and sensitivity specialized for their respective cellular compartments, and this increased the accuracy of subcellular pH measurements based on extensive preliminary tests. Both probes were calibrated under the same experimental conditions, and thus enable reliable and standardized comparisons of pH values across cytosolic and vacuolar compartments. Calibration curves for both cytosolic and vacuolar pH measurements were constructed using samples equilibrated with a calibration buffer containing nigericin (30 µM) and 110 mM K+ to eliminate intracellular pH gradients (electronic supplementary material, figures S1–S4).

### (c) Measurement of reactive oxygen species

Intracellular ROS distributions were visualized using the fluorescent probe 5(6)-carboxy-2′,7′-dichlorofluorescein diacetate (DCFDA) [40]. Fungal samples were incubated with 10 µM DCFDA in the dark at 25°C for 20 min and then washed three times with PBS to remove excess dye before imaging. Confocal microscopy was then performed using a Leica Stellaris 8 confocal laser scanning microscope with a 40× water immersion objective. ROS fluorescence was recorded at 530 nm (±20 nm) after excitation at 504 nm. The average intensity of the DCFDA signal was collected in regions of interest in the vacuole and cytosol of both hyphal and gongylidium cells. We quantified ROS using ImageJ software, quantifying fluorescence intensity measurements from the cytosol and vacuole of gongylidium and hyphal cells. Fluorescence values from stained samples were background-corrected by subtracting the mean autofluorescence measured in the corresponding unstained controls.

### (d) Statistical analysis

We used R [41] to perform all statistical analyses. We provide mean values in the text ±1 s.d. We performed two separate two-way ANOVAs to test for the differences in each of the response variables, pH and ROS, between the independent variables cell type (hyphal cell, gongylidium cell), compartment (cytosol, vacuole) and the cell type × compartment interaction. We log_10_-transformed ROS values prior to the analysis to meet the assumptions of normality and homogeneity of variance. We note that subsequent interaction effects reflected multiplicative relationships between compartment and cell type. For both ANOVA tests, significant main effects were followed by *post hoc* Tukey tests.

## 3. Results

### (a) Testing for pH differences coinciding with tissue specialization

**Figure 1.**
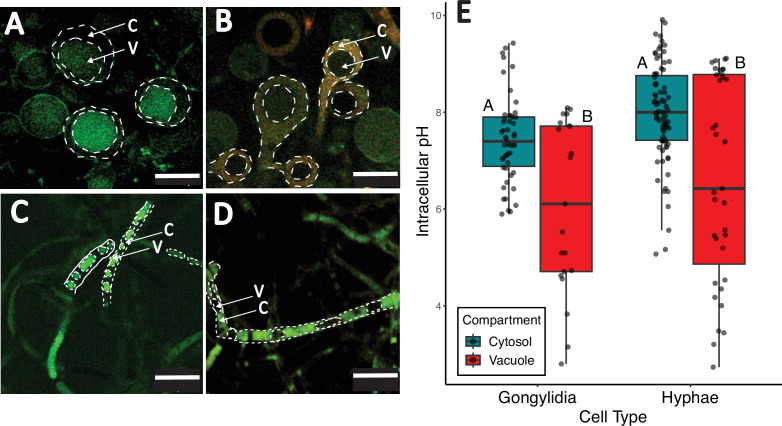
Comparing pH across cell types and cell compartments in the fungus cultivated by leafcutter ants. (A) Gongylidium cell vacuolar pH measured using pHrodo staining. (B) Gongylidium cell cytosolic pH measured using carboxy SNARF-1AM staining. SNARF-1 was excited at 543 nm, and fluorescence emission was collected at 585 nm (displayed in red; more acidic pH) and 640 nm (displayed in green; more neutral to basic pH). The ratio of these two emission intensities was used to estimate relative cytosolic pH. (C) Hyphal cell vacuolar pH measured using pHrodo staining. (D) Hyphal cell cytosolic pH measured using carboxy SNARF-1AM staining with the same colour coding as in panel (B). In all images, C indicates cytosol and V indicates vacuoles. Scale bars: 40 µm in panels (A) and (B), 20 µm in panel (C) and 30 µm in panel (D). (E) Box plot comparing pH across cell types and subcellular compartments. The horizontal line within each box indicates the median pH value; the top and bottom of each box represent the first and third quartiles, respectively, and whiskers extend to 1.5× the interquartile range. Jittered raw data values are overlaid atop the box plots. Within cell types, different letters indicate significant pairwise differences (Tukey–Kramer test, *p* < 0.05); identical letters within a cell type denote no significant difference.

Imaging of pH in vacuoles and cytosol of gongylidium cells (figure 1A,B) and hyphal cells (figure 1C,D) indicated that vacuolar pH specialization is not a key feature of gongylidium tissue differentiation. First, while pH was lower in vacuoles (6.42 ± 2.00) than in the surrounding cytosol (7.78 ± 1.01) (compartment: *F*_1,181_ = 39.08; *p* < 0.00001), this effect was consistent for both cell types (figure 1E). To this point, a significant difference in pH between cell types (cell type: *F*_1,181_ = 7.26; *p* = 0.008) was driven by a substantially larger pairwise difference between gongylidium vacuoles and hypha cytosol (*post hoc* Tukey, *p* < 0.00001) than between hypha vacuoles and gongylidium cytosol (*post hoc* Tukey, *p* = 0.04) (figure 1E).

Second, the consistent pH compartmentalization effects observed across both cell types (cell type × compartment: *F*_1,181_ = 0.003; *p* = 0.954) indicate a lack of subcellular specialization of pH in gongylidium cells (figure 1E). Specifically, cytosolic pH did not differ significantly between gongylidium cells (7.44 ± 0.90) and hyphal cells (7.99 ± 1.03) (*post hoc* Tukey, *p* = 0.121) and vacuolar pH did not differ significantly between gongylidium cells (6.07 ± 1.77) and hyphal cells (6.64 ± 2.12) (*post hoc* Tukey, *p* = 0.425) (figure 1E).

### (b) Testing for reactive oxygen speciesdifferences coinciding with tissue specialization

**Figure 2.**
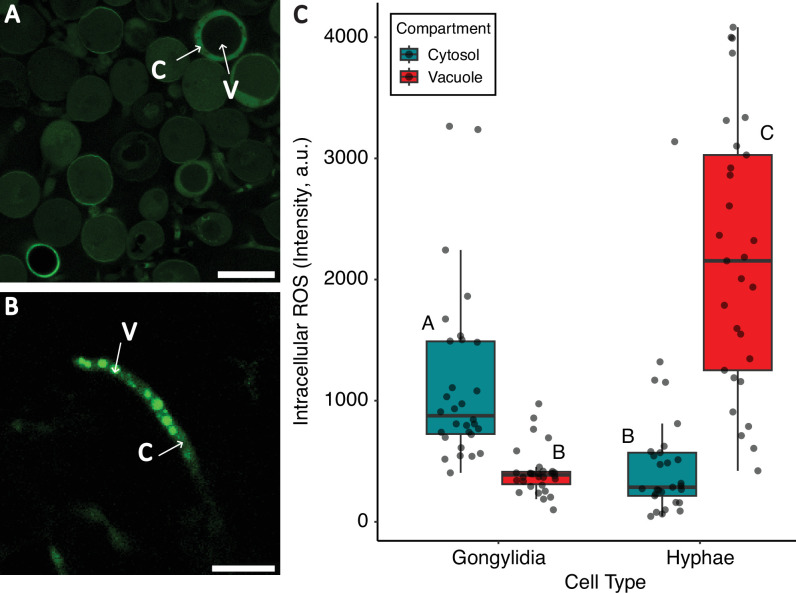
Comparing ROS levels across cell types and cell compartments in the fungus cultivated by leafcutter ants. Representative ROS levels in the cytosol (indicated by 'C') and vacuoles (indicated by 'V') of (A) gongylidium cells and (B) hyphal cells measured using confocal imaging following DCFDA staining. Scale bars in both panels indicate 50 µm. (C) Box plot comparing ROS across cell types and subcellular compartments. The box plot elements are as described in figure 1. Different letters indicate significant pairwise differences (Tukey test, *p* < 0.05).

In contrast to the pH results, imaging of ROS concentrations in vacuoles and surrounding cytosol revealed fundamental differences in compartmentalization strategies between gongylidia (figure 2A) and hyphae (figure 2B). First, ROS values differed both across cell types (*F*_1,114_ = 3.97; *p* = 0.049) and across compartments (*F*_1,114_ = 8.90; *p* = 0.003) (figure 2C). Second, a significant cell type by compartment interaction (*F*_1,114_ = 124.30; *p* < 0.00001) is supported by *post hoc* Tukey–Kramer tests indicated that ROS levels in these compartments differed in opposite directions (figure 2C). Thus, while hyphal vacuoles stored ROS, gongylidia vacuoles tended to exclude them.

## 4. Discussion

Gongylidia are the cellular interface where the farming efforts of ants become realized nutritional rewards, and they are delivery capsules that safely transport functional metabolites to ant digestive tracts. These cells are thus hub-like centres of multimodal transmission whose sophistication we are just beginning to grasp. Yet, the underlying cellular adaptations mediating these derived functions have remained poorly understood. Our subcellular imaging results indicate that gongylidium vacuoles gained the function of excluding ROS rather than sequestering and degrading them. Thus, gongylidium cells compartmentalize potentially harmful molecules in the opposite way to undifferentiated hyphal cells. To the extent that this change in vacuolar ROS concentration promotes mutualistic trade, it may be crucial for the eco-evolutionary success of leafcutter farming systems.

In contrast to the vacuolar results, ROS levels were elevated in gongylidium cytosol relative to hyphal cytosol. While this may simply reflect that cytosol is the only ROS reservoir upon being excluded from gongylidium vacuoles, this ROS pattern may also have functional significance. For instance, the accumulation of cytosolic ROS may play a role in elevated expression of autophagy pathways putatively linked to gongylidium cell differentiation [26]. ROS can also more generally promote baseline cell functions including regulation of cytosolic antioxidant pathways that can maintain redox homeostasis during nutrient processing [42] and regulation of cell differentiation by activating downstream genes [35].

Vacuoles of both cell types had similarly low pH relative to the surrounding cytosol. We further observed substantial variation in pH across vacuoles in both cell types. Vacuolar pH is known to vary with vacuole function and metabolic state, for example, acidifying during autophagy or storage, and alkalinizing during mobilization of stored compounds [43,44]. Regardless of the mechanism, these results imply that the ant-targeted metabolites stored within gongylidium vacuoles do not require specialized pH conditions.

The *in vitro* snapshots of subcellular ROS differences we observe between gongylidia and hyphae set the stage to next consider the mechanisms that dynamically trigger this cell type differentiation. This is because these mechanisms regulate the fungal cultivar’s edible yield. For example, if leafcutter ants can directly trigger gongylidium cell differentiation by depositing a specific metabolite that induces this process, they could precisely regulate cultivar productivity. Alternatively, if the fungus has complete regulatory control over gongylidium cell differentiation, ant farmers could only indirectly promote this process by regulating the farming environment. We hypothesize that ROS compartmentalization supports an endogenous fungal-control mechanism. This is because ROS can initiate cell differentiation by modulating autophagy, energy metabolism and organelle remodelling [45]. Moreover, elevated cytosolic ROS levels may trigger mitochondrial signalling cascades that activate autophagic degradation of organelles and yield metabolic by-products whose influx into gongylidium vacuoles initiates cellular swelling—as has been observed in plant cells [46]. Testing this hypothesis can help understand mechanisms of symbiotic stability at cellular scales where animals and fungi interact [45,46].

The subcellular differentiation processes yielding gongylidium cells are also interesting because they may be unique among fungi. Hyphae of most filamentous fungi are repetitive modular cellular compartments, and cell type differentiation is typically limited to fruiting bodies [47]. Some have hypothesized that gongylidia are derived from cystidia, the sterile and often swollen cells found on spore-bearing surfaces of some basidiomycete fungal relatives of *L. gongylophorus* [8]. Even so, the subcellular modifications we observed in gongylidia could probably only have evolved because the fungus has been strictly vertically transmitted and associated with leafcutter farming systems for millions of years [5,48]. Gongylidia are thus key to understanding the selection pressures mediating coevolutionary crop domestication.

## Data Availability

Data and code for reproducing the analyses and figures is submitted as electronic supplementary material. Supplementary material is available online [49].

## References

[1] Kiers TE, Palmer TM, Ives AR, Bruno JF, Bronstein JL. 2010 Mutualisms in a changing world: an evolutionary perspective. Ecol. Lett. **13**, 1459–1474. (10.1111/j.1461-0248.2010.01538.x) 20955506

[2] De Fine Licht HH, Boomsma JJ, Tunlid A. 2014 Symbiotic adaptations in the fungal cultivar of leaf-cutting ants. Nat. Commun. **5**, 5675. (10.1038/ncomms6675) 25435021

[3] Leal-Dutra CA, Vizueta J, Baril T, Kooij PW, Rødsgaard-Jørgensen A, Conlon BH, Croll D, Shik JZ. 2024 Genomic signatures of domestication in a fungus obligately farmed by leafcutter ants. Mol. Biol. Evol. **41**, msae197. (10.1093/molbev/msae197) 39288321 PMC11451569

[4] Hölldobler B, Wilson EO. 2011 The leafcutter ants: civilization by instinct. New York, NY: W. W. Norton & Company.

[5] Schultz TR *et al*. 2024 The coevolution of fungus–ant agriculture. Science **386**, 105–110. (10.1126/science.adn7179) 39361762

[6] Wirth R, Herz H, Ryel RJ, Beyschlag W, Hölldobler B. 2003 Herbivory of leaf-cutting ants. Berlin, Heidelberg, Germany: Springer Berlin Heidelberg. (10.1007/978-3-662-05259-4)

[7] Bolander M, Andersen JE, Conlon BH, Arnan X, Michelsen A, Shik JZ. 2023 Reciprocal nutritional provisioning between leafcutter ants and their fungal cultivar mediates performance of symbiotic farming systems. Funct. Ecol. **37**, 3079–3090. (10.1111/1365-2435.14437)

[8] Mueller UG, Schultz TR, Currie CR, Malloch D. 2001 The origin of the attine ant–fungus mutualism. Q. Rev. Biol. **76**, 169–197. (10.1086/393867) 11409051

[9] Shik JZ, Rytter W, Arnan X, Michelsen A. 2018 Disentangling nutritional pathways linking leafcutter ants and their co‐evolved fungal symbionts using stable isotopes. Ecology **99**, 1999–2009. (10.1002/ecy.2431) 30067862 PMC6174977

[10] Currie CR, Poulsen M, Mendenhall J, Boomsma JJ, Billen J. 2006 Coevolved crypts and exocrine glands support mutualistic bacteria in fungus-growing ants. Science **311**, 81–83. (10.1126/science.1119744) 16400148

[11] Birkenfeld V, Gorb SN, Krings W. 2024 Mandible elemental composition and mechanical properties from distinct castes of the leafcutter ant Atta laevigata (Attini; Formicidae). Interface Focus **14**, 20230048. (10.1098/rsfs.2023.0048) 38618230 PMC11008964

[12] Agavekar G, Suzuki Y, Suenaga M, Pacheco-Leiva M, Hiller M, Pinto-Tomas AA, Myers E, Economo EP. 2025 A haplotype-resolved chromosome-scale genome assembly and annotation for the leafcutter ant, Acromyrmex octospinosus. Genome Biol. Evol. **17**, evaf047. (10.1093/gbe/evaf047) 40066805 PMC11926981

[13] Fernández-Marín H, Zimmerman JK, Nash DR, Boomsma JJ, Wcislo WT. 2009 Reduced biological control and enhanced chemical pest management in the evolution of fungus farming in ants. Proc. R. Soc. B **276**, 2263–2269. (10.1098/rspb.2009.0184) PMC2677613 19324734

[14] Grell MN, Linde T, Nygaard S, Nielsen KL, Boomsma JJ, Lange L. 2013 The fungal symbiont of Acromyrmex leaf-cutting ants expresses the full spectrum of genes to degrade cellulose and other plant cell wall polysaccharides. BMC Genom. **14**, 928. (10.1186/1471-2164-14-928) PMC3880420 24373541

[15] Moller IE, De Fine Licht HH, Harholt J, Willats WGT, Boomsma JJ. 2011 The dynamics of plant cell-wall polysaccharide decomposition in leaf-cutting ant fungus gardens. PLoS One **6**, e17506. (10.1371/journal.pone.0017506) 21423735 PMC3053354

[16] Schiøtt M, De Fine Licht HH, Lange L, Boomsma JJ. 2008 Towards a molecular understanding of symbiont function: identification of a fungal gene for the degradation of xylan in the fungus gardens of leaf-cutting ants. BMC Microbiol. **8**, 40. (10.1186/1471-2180-8-40) 18307762 PMC2291056

[17] Conlon BH, O’Tuama D, Michelsen A, Crumière AJJ, Shik JZ. 2022 A fungal symbiont converts provisioned cellulose into edible yield for its leafcutter ant farmers. Biol. Lett. **18**, 20220022. (10.1098/rsbl.2022.0022) 35440234 PMC9019514

[18] De Fine Licht HH, Schiøtt M, Rogowska-Wrzesinska A, Nygaard S, Roepstorff P, Boomsma JJ. 2013 Laccase detoxification mediates the nutritional alliance between leaf-cutting ants and fungus-garden symbionts. Proc. Natl Acad. Sci. USA **110**, 583–587. (10.1073/pnas.1212709110) 23267060 PMC3545816

[19] Rønhede S, Boomsma JJ, Rosendahl S. 2004 Fungal enzymes transferred by leaf-cutting ants in their fungus gardens. Mycol. Res. **108**, 101–106. (10.1017/s0953756203008931) 15035511

[20] Nygaard S *et al*. 2016 Reciprocal genomic evolution in the ant–fungus agricultural symbiosis. Nat. Commun. **7**, 12233. (10.1038/ncomms12233) 27436133 PMC4961791

[21] Kooij PW, Aanen DK, Schiøtt M, Boomsma JJ. 2015 Evolutionarily advanced ant farmers rear polyploid fungal crops. J. Evol. Biol. **28**, 1911–1924. (10.1111/jeb.12718) 26265100 PMC5014177

[22] Chalhoub B *et al*. 2014 Early allopolyploid evolution in the post-neolithic Brassica napus oilseed genome. Science **345**, 950–953. (10.1126/science.1253435) 25146293

[23] Khadempour L *et al*. 2021 From plants to ants: fungal modification of leaf lipids for nutrition and communication in the leaf-cutter ant fungal garden ecosystem. mSystems **6**, e01307-20. (10.1128/mSystems.01307-20) 33758033 PMC8547007

[24] De Fine Licht HH, Schiøtt M, Mueller UG, Boomsma JJ. 2010 Evolutionary transitions in enzyme activity of ant fungus gardens. Evolution **64**, 2055–2069. (10.1111/j.1558-5646.2010.00948.x) 20067517

[25] Quinlan RJ, Cherrett JM. 1979 The role of fungus in the diet of the leaf‐cutting ant Atta cephalotes (L.). Ecol. Entomol. **4**, 151–160. (10.1111/j.1365-2311.1979.tb00570.x)

[26] Leal-Dutra CA, Yuen LM, Guedes BAM, Contreras-Serrano M, Marques PE, Shik JZ. 2023 Evidence that the domesticated fungus Leucoagaricus gongylophorus recycles its cytoplasmic contents as nutritional rewards to feed its leafcutter ant farmers. IMA Fungus **14**, 19. (10.1186/s43008-023-00126-5) 37715276 PMC10503033

[27] Veses V, Richards A, Gow NA. 2008 Vacuoles and fungal biology. Curr. Opin. Microbiol. **11**, 503–510. (10.1016/j.mib.2008.09.017) 18935977

[28] Kooij PW, Rogowska-Wrzesinska A, Hoffmann D, Roepstorff P, Boomsma JJ, Schiøtt M. 2014 Leucoagaricus gongylophorus uses leaf-cutting ants to vector proteolytic enzymes towards new plant substrate. ISME J. **8**, 1032–1040. (10.1038/ismej.2013.231) 24401858 PMC3996701

[29] Schiøtt M, Rogowska-Wrzesinska A, Roepstorff P, Boomsma JJ. 2010 Leaf-cutting ant fungi produce cell wall degrading pectinase complexes reminiscent of phytopathogenic fungi. BMC Biol. **8**, 156. (10.1186/1741-7007-8-156) 21194476 PMC3022778

[30] Hesse SJA, Ruijter GJG, Dijkema C, Visser J. 2002 Intracellular pH homeostasis in the filamentous fungus Aspergillus niger. Eur. J. Biochem. **269**, 3485–3494. (10.1046/j.1432-1033.2002.03042.x) 12135488

[31] Martins MP, Martinez-Rossi NM, Sanches PR, Gomes EV, Bertolini MC, Pedersoli WR, Silva RN, Rossi A. 2019 The pH signaling transcription factor PAC-3 regulates metabolic and developmental processes in pathogenic fungi. Front. Microbiol. **10**, 2076. (10.3389/fmicb.2019.02076) 31551996 PMC6738131

[32] Veiter L, Rajamanickam V, Herwig C. 2018 The filamentous fungal pellet—relationship between morphology and productivity. Appl. Microbiol. Biotechnol. **102**, 2997–3006. (10.1007/s00253-018-8818-7) 29473099 PMC5852183

[33] Juan CA, Pérez de la Lastra JM, Plou FJ, Pérez-Lebeña E. 2021 The chemistry of reactive oxygen species (ROS) revisited: outlining their role in biological macromolecules (DNA, lipids and proteins) and induced pathologies. Int. J. Mol. Sci. **22**, 4642. (10.3390/ijms22094642) 33924958 PMC8125527

[34] Warris A, Ballou ER. 2019 Oxidative responses and fungal infection biology. Semin. Cell Dev. Biol. **89**, 34–46. (10.1016/j.semcdb.2018.03.004) 29522807

[35] Schieber M, Chandel NS. 2014 ROS function in redox signaling and oxidative stress. Curr. Biol. **24**, R453–R462. (10.1016/j.cub.2014.03.034) 24845678 PMC4055301

[36] Branco S, Schauster A, Liao H, Ruytinx J. 2022 Mechanisms of stress tolerance and their effects on the ecology and evolution of mycorrhizal fungi. New Phytol. **235**, 2158–2175. (10.1111/nph.18308) 35713988

[37] Wahab A, Muhammad M, Munir A, Abdi G, Zaman W, Ayaz A, Khizar C, Reddy SPP. 2023 Role of arbuscular mycorrhizal fungi in regulating growth, enhancing productivity, and potentially influencing ecosystems under abiotic and biotic stresses. Plants **12**, 3102. (10.3390/plants12173102) 37687353 PMC10489935

[38] Venn AA, Tambutté E, Lotto S, Zoccola D, Allemand D, Tambutté S. Imaging intracellular pH in a reef coral and symbiotic anemone. Proc. Natl Acad. Sci. USA **106**, 16574–16579. (10.1073/pnas.0902894106) PMC2757848 19720994

[39] Ogawa M, Kosaka N, Regino CAS, Mitsunaga M, Choyke PL, Kobayashi H. 2010 High sensitivity detection of cancer in vivo using a dual-controlled activation fluorescent imaging probe based on H-dimer formation and pH activation. Mol. Biosyst. **6**, 888. (10.1039/b917876g) 20567775 PMC3464101

[40] Pogue AI, Jones BM, Bhattacharjee S, Percy ME, Zhao Y, Lukiw WJ. 2012 Metal-sulfate induced generation of ROS in human brain cells: detection using an isomeric mixture of 5- and 6-carboxy-2′,7′-dichlorofluorescein diacetate (carboxy-DCFDA) as a cell permeant tracer. Int. J. Mol. Sci. **13**, 9615–9626. (10.3390/ijms13089615) 22949820 PMC3431818

[41] R Core Team. 2023 R: a language and environment for statistical computing. Vienna, Austria: R Foundation for Statistical Computing. See http://www.R-project.org/.

[42] Görlach A, Dimova EY, Petry A, Martínez-Ruiz A, Hernansanz-Agustín P, Rolo AP, Palmeira CM, Kietzmann T. 2015 Reactive oxygen species, nutrition, hypoxia and diseases: problems solved? Redox Biol. **6**, 372–385. (10.1016/j.redox.2015.08.016) 26339717 PMC4565025

[43] Klionsky DJ, Herman PK, Emr SD. 1990 The fungal vacuole: composition, function, and biogenesis. Microbiol. Rev. **54**, 266–292. (10.1128/mr.54.3.266-292.1990) 2215422 PMC372777

[44] Li SC, Kane PM. 2009 The yeast lysosome-like vacuole: endpoint and crossroads. Biochim. Biophys. Acta **1793**, 650–663. (10.1016/j.bbamcr.2008.08.003) 18786576 PMC2906225

[45] Craige SM, Mammel RK, Amiri N, Willoughby OS, Drake JC. 2024 Interplay of ROS, mitochondrial quality, and exercise in aging: potential role of spatially discrete signaling. Redox Biol. **77**, 103371. (10.1016/j.redox.2024.103371) 39357424 PMC11474192

[46] Schmidt R, Kunkowska AB, Schippers JHM. 2016 Role of reactive oxygen species during cell expansion in leaves. Plant Physiol. **172**, 2098–2106. (10.1104/pp.16.00426) 27794099 PMC5129704

[47] Teichert I, Nowrousian M, Pöggeler S, Kück U. 2014 The filamentous fungus Sordaria macrospora as a genetic model to study fruiting body development. Adv. Genet. **87**, 199–244. (10.1016/B978-0-12-800149-3.00004-4) 25311923

[48] Branstetter MG, Ješovnik A, Sosa-Calvo J, Lloyd MW, Faircloth BC, Brady SG, Schultz TR. 2017 Dry habitats were crucibles of domestication in the evolution of agriculture in ants. Proc. R. Soc. B **284**, 20170095. (10.1098/rspb.2017.0095) PMC5394666 28404776

[49] Stelate A, Shik JZ. 2025 Supplementary material from: Evidence of cellular adaptations in a fungal cultivar promoting resource exchange with leafcutter ant farmers. Figshare. (10.6084/m9.figshare.c.8054266)41027472

